# The use of social care services by individuals aged 50 and over diagnosed with colorectal cancer in Scotland: a linked data study.

**DOI:** 10.23889/ijpds.v7i3.2017

**Published:** 2022-08-25

**Authors:** Elizabeth Lemmon, Alasdair Rutherford, Peter Hall, David Bell, David Henderson, Amy Downing, Steve Clark

**Affiliations:** 1 University of Edinburgh, Edinburgh Health Economics; 2 University of Stirling; 3 University of Edinburgh; 4 University of Leeds; 5 UK Colorectal Intelligence Hub Patient Public Group

The objective of this paper is to explore how a colorectal cancer (CRC) diagnosis influences an individual’s use of social care services in Scotland. In particular, we aim to compare this use to individual’s diagnosed with other cancers and to those without a cancer diagnosis.

We use a linked health and social care dataset of the Scottish population aged 50 and over in the financial year 2015/16. Our approach involves several methods to estimate the effect of a CRC diagnosis on social care use. Firstly, we conduct difference in means tests. Secondly, we estimate two-part models of the utilisation of social care for the CRC, other cancer and non-cancer groups. Lastly, we use propensity score matching. Models are estimated for those diagnosed during 2015/16 and for those diagnosed during 2015/16 or with an historical diagnosis.

Preliminary results reveal that the likelihood of receipt of social care services is higher for those diagnosed with CRC compared to those without a cancer diagnosis. Further, individuals with a non-CRC cancer diagnosis are more likely to receive social care services compared to those without a cancer diagnosis, but they are less likely to receive social care compared to those with a CRC diagnosis. Further, the likelihood of care receipt for the CRC and other cancer group increases as the number of years since a cancer diagnosis increases. In terms of the number of services received, CRC patients and those with other types of cancer receive fewer services when compared to those without a cancer diagnosis.

Our paper has demonstrated that a CRC diagnosis has a significant impact on an individual’s use of social care services. However, conditional on receiving social care, the number of services received is lower for individuals with CRC. Further research is required to understand whether the needs of those individuals are being met.

